# Anthocyanins and reactive oxygen species: a team of rivals regulating plant development?

**DOI:** 10.1007/s11103-023-01362-4

**Published:** 2023-06-23

**Authors:** João Victor A. Cerqueira, Moab T. de Andrade, Diego D. Rafael, Feng Zhu, Samuel V. C. Martins, Adriano Nunes-Nesi, Vagner Benedito, Alisdair R. Fernie, Agustin Zsögön

**Affiliations:** 1 Departamento de Biologia Vegetal, Universidade Federal de Viçosa, Viçosa, MG 36570-900 Brazil; 2 Max-Planck-Institute for Molecular Plant Physiology, 14476 Potsdam, Germany; 3 Key Laboratory of Horticultural Plant Biology, Ministry of Education, National R&D Center for Citrus Preservation, Huazhong Agricultural University, Wuhan, 430070 China; 4 Division of Plant and Soil Sciences, West Virginia University, Morgantown, WV 26506 USA

**Keywords:** Antioxidants, Phenolic compounds, Flavonoids, ROS, Cell signalling, Stress response

## Abstract

Anthocyanins are a family of water-soluble vacuolar pigments present in almost all flowering plants. The chemistry, biosynthesis and functions of these flavonoids have been intensively studied, in part due to their benefit for human health. Given that they are efficient antioxidants, intense research has been devoted to studying their possible roles against damage caused by reactive oxygen species (ROS). However, the redox homeostasis established between antioxidants and ROS is important for plant growth and development. On the one hand, high levels of ROS can damage DNA, proteins, and lipids, on the other, they are also required for cell signaling, plant development and stress responses. Thus, a balance is needed in which antioxidants can remove excessive ROS, while not precluding ROS from triggering important cellular signaling cascades. In this article, we discuss how anthocyanins and ROS interact and how a deeper understanding of the balance between them could help improve plant productivity, nutritional value, and resistance to stress, while simultaneously maintaining proper cellular function and plant growth.

## Key message

The balance between anthocyanins and ROS could be manipulated to improve plant productivity, nutritional value, and resistance to stress.

## Introduction

Anthocyanins are polyphenols of the flavonoid class of specialized plant metabolites (Smeriglio et al. 2016). They consist of anthocyanidin skeletons with carbohydrate side chains. It is currently unclear which selective pressures have caused anthocyanins to become almost ubiquitous among plants (Wen et al. 2020). Indeed, the presence of red–violet pigments in all land plants is considered a prime example of convergent evolution (Piatkowski et al. 2020). The anthocyanin biosynthetic pathway did not evolve until the emergence of seed plants, while the liverworts, mosses, lycophytes, and ferns only have “anthocyanin-like” red–violet pigments (Piatkowski et al. 2020). Specifically, the emergence of the flavanone-3-hydroxylase enzyme (F3H) was the key event in the production of the flavonoid backbone and the enormous variety of flavonoids and anthocyanins in seed plants, possibly originating from an ancestor transitional form of a flavone synthase I (FNS I), which is widely distributed in more basal land plants (Li et al. 2020; Piatkowski et al. 2020). In terms of function, the roles of anthocyanin in plants as a photo-attenuator (Zheng et al. 2021) metal–chelator (Macar et al. 2020; Xie et al. 2018) and antioxidant (Trojak and Skowron 2017), have been extensively demonstrated. Another factor that supports the importance of these pigments to the plant is the considerable energetic cost of their biosynthesis, with each phenol ring having an ΔrH° ≅ 165 kJ (Parks et al. 1954). The anthocyanin molecule has a C15 (C6-C3-C6) structure, where each C6 is a phenol ring. Thus, each anthocyanin molecule requires an investment of more than 330 kJ to construct.

There is growing interest in elevating the levels of anthocyanins in edible plants for dietary purposes (Butelli et al. 2008; Gonzali and Perata 2020), mainly due to their role as scavengers of reactive oxygen species (ROS). ROS can trigger degradation of proteins, lipid peroxidation and DNA damage (Smirnoff and Arnaud 2019), often leading to cell senescence and death (Cheung and Vousden 2022). On the other hand, an equilibrium between antioxidants and ROS is essential for plant development despite their opposite functions (Schippers et al. 2016). In this article, we provide a brief overview of anthocyanins and ROS and discuss how knowledge about the balance between the levels of these two classes of chemicals can be harnessed for agricultural benefits.

### Anthocyanins

The term “anthocyanins” means “blue flowers”, however they can be found in other plant organs and display shades of pink, red, purple and blue depending on the chemical structure, intravacuolar pH, co-pigments and interacting metal ions (Kallam et al. 2017). Here, we summarize the current knowledge of anthocyanin chemistry, their biosynthesis and metabolism, and finally their function as antioxidants.

Anthocyanins are water-soluble flavonoids composed of three elements: an anthocyanidin backbone decorated with sugar moieties and, occasionally, acyl-conjugates (Sasaki et al. 2014; Stommel et al. 2009). Basically, an anthocyanidin is a structure composed of two benzyl rings connected by a heterocyclic ring. Anthocyanidin glycosylation enhances water solubility and acylation increases stability (Mazza et al. 2004; Smeriglio et al. 2016). Changes in vacuolar pH alter the color of anthocyanins due to variation in the resonance structure produced by electronic transitions (Smeriglio et al. 2016; Wrolstad et al. 2005). Resonance also helps explain the redox properties of anthocyanins, which are relevant for their antioxidant capacity (Smeriglio et al. 2016). The biosynthesis of anthocyanins is increased by environmental stresses such as high irradiance, cold, drought and nutrient deficiency, especially nitrogen and phosphorus (Chalker-Scott 1999; Nakabayashi et al. 2014; Oren-Shamir 2009). Anthocyanin degradation seems to also be stimulated under stress, and at least under drought, increases have been reported in the activity of vacuolar β-glucosidase, the key enzyme involved in the first step of anthocyanin degradation (Wang et al. 2011). By the same token, stress-responsive hormones, like abscisic acid (ABA) and jasmonates (JA), also induce anthocyanins accumulation (Cotado et al. 2018; Li et al. 2019a, b; Zhou et al. 2009). A convergence point between abiotic stress and anthocyanins biosynthesis is that both are activated by ROS accumulation (Nakabayashi et al. 2014). Despite these findings, the cost-benefit ratio to produce these pigments remains unclear (Lo Piccolo et al. 2020). Interestingly, in the Caryophyllales order anthocyanins are absent, and the alkaloid betacyanins are the replacement pigment taking over their function (Jain and Gould 2015; Stafford 1994). Their accumulation also depends on environmental factors but anthocyanins and betacyanins never occur together in the same plant and little is known about the molecular basis of this mutual exclusion (Sakuta 2014). Phylogenetic analyses suggests that this is a legacy of the single and early origin of betacyanin pigmentation in the evolutionary history of the order, which was lost in the anthocyanin lineages (Brockington et al. 2015; Polturak and Aharoni 2018).

**Fig. 1:**
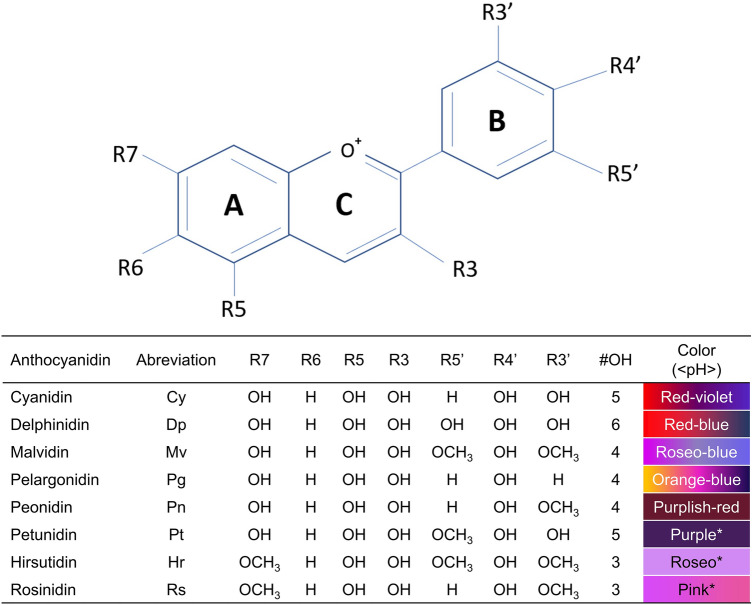
Molecular structure of anthocyanins. The C6-C3-C6 show seven substitution positions that are enumerated (R3, R4, R5, R6, R7, R3’, R4’ and R5’). The color and antioxidant capacity of anthocyanins depend directly on these positions. *Coloration derived from the flowers that contain the compound, as *Catharanthus roseus*, *Primula rosea* and *Petunia* sp

The chemical structure of flavonoids has a direct impact on their ability to scavenge different types of ROS (Dimitrić Marković et al. 2014). Zhang et al. (2015) demonstrated that the presence of OH groups in the B-ring influences the antioxidant activity of the molecule (Fig. 1). Glycosylation decreases the scavenger activity compared to the original aglycones structures, thus minimizing their proton donating and metal chelating abilities (Zhao et al. 2014). Other B-ring substitutes can alter the antioxidant activity. Among the six types of natural anthocyanidins, pelargonidin has four OH groups and showed the lowest antioxidant activity whereas delphinidin, which has six OH groups, showed the highest activity (Rahman et al. 2006).

Biochemically, anthocyanins have been described as compounds that prevent the oxidation by scavenging free radicals and reducing the oxidative stress, given that anthocyanins act as H-atom donor or as single electron transfer (Einbond et al. 2004). Also, the main structure of anthocyanins, the anthocyanin chalcones and the quinoidal base with double bonds conjugated to the keto group are efficient antioxidants (Smeriglio et al. 2016). The antioxidant capacity of anthocyanins has already been demonstrated *in planta*. In Arabidopsis, flavonol and anthocyanin over-accumulators, such as transgenic lines overexpressing *ANTHOCYANIN PIGMENT 1* (*PAP1/MYB75*) or *PRODUCTION OF FLAVONOL GLYCOSIDES 1* (*MYB12/PFG1*) showed higher free radical scavenger activities than the wild type (Nakabayashi et al. 2014). Corroborating this increased antioxidant capacity, these cyanic lines showed three-fold higher survival rates in methyl viologen-induced oxidative stress compared to wild-type plants (Nakabayashi et al. 2014). On the flip side, Arabidopsis mutants deficient in anthocyanins, such as *transparent testa* (*tt*), showed greater levels of endogenous ROS and lower antioxidant capacity under high-irradiance stress (Xu et al. 2017).

**Fig. 2:**
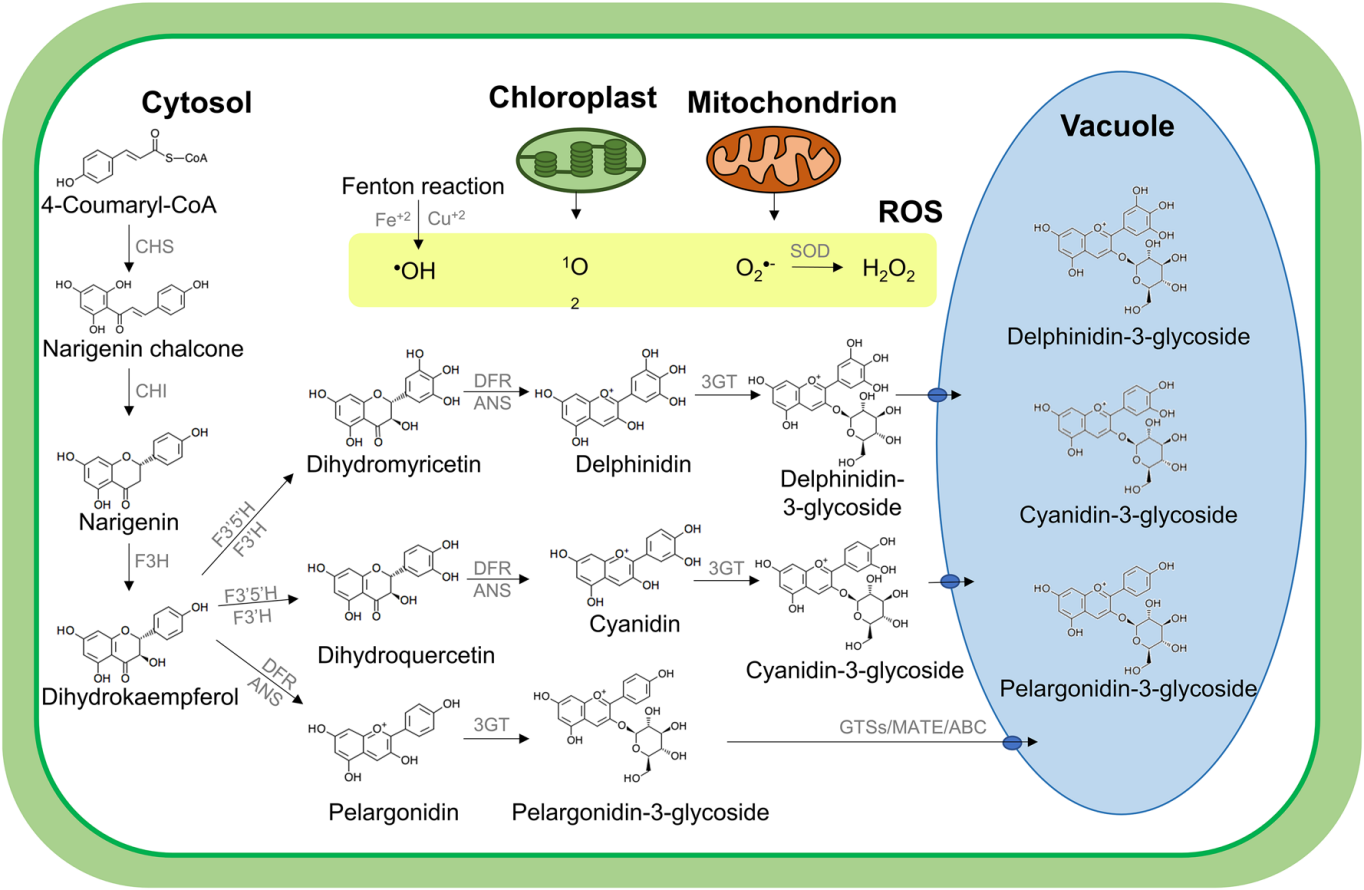
Anthocyanins biosynthesis, transport and ROS production in different compartments of a standard plant cell. *CHS* chalcone synthase; *CHI* chalcone isomerase; *F3H* flavanone 3-dioxygenase; *F3′5′H* flavonoid 3′,5′ hydroxylase; *F3′H* flavanone 3′-hydroxylase; *DFR* dihydroflavonol 4-reductase; *ANS* anthocyanidin synthase; *3GT* anthocyanidin 3-O-glucosyltransferase; *GSTs* glutathione S-transferases; *MATE* Multidrug and toxic compound extrusion; *ABC* ATP-binding cassette transporters; *SOD* superoxide dismutase

The anthocyanin biosynthesis pathway is highly conserved in plants (Chen et al. 2019) (Fig. 2). The expression of ‘early biosynthesis genes’ (EBGs: *CHS*, *CHI*, *F3H* and *F3’H*) are regulated directly by R2R3-MYB transcription factors while the ‘late biosynthesis genes’ (LBGs: *DFR*, *LDOX*, *ANS* and *UF3GT*) and membrane transporters (*TT12* and *ABCC*) are controlled by the MBW (MYB-bHLH-WD40) transcriptional activation complex (Chaves-Silva et al. 2018; Chen et al. 2019). Many studies demonstrated that R2R3-MYB transcription factors play critical roles against biotic and abiotic stresses (Ai et al. 2018; He et al. 2020; Wei et al. 2017). A simplistic model is that while stresses disrupt ROS scavenging mechanisms and promotes cell damage by oxidative stress (Miller et al. 2010; Xu and Rothstein 2018), they also trigger anthocyanin biosynthesis for increased antioxidant protection (Chalker-Scott 1999). However, the regulation of the biosynthesis of anthocyanins and phenylpropanoids in general is complex and the effect of the MBW complex can be either positive (Broeckling et al. 2016; Li et al. 2020) or negative (Dong et al. 2020; Xiang et al. 2019), depending on the transcription factors participating in its composition (Chaves-Silva et al. 2018).

### Reactive oxygen species (ROS)

Molecular oxygen (O_2_) is a stable molecule with two unpaired electrons with parallel spin in a degenerated π orbital. As a consequence, O_2_ accepts one electron at a time, which generates ROS (Gill and Tuteja 2010) (Table 1). Some organelles have high rates of oxygen reduction, making them highly vulnerable to oxidative stress, such as mitochondria due to respiration, chloroplasts due to photosynthesis, and peroxisomes and glyoxysomes due to photorespiration and fatty acid β-oxidation, respectively (Daiber and Münzel 2015; Zurbriggen et al. 2010). The various types of ROS are produced through different pathways in each organelle and their concentrations possibly help the cell to recognize regions of physiological disturbance during stressful conditions (Singh et al. 2019). In mitochondria, the first ROS produced is the radical superoxide (O_2_^·−^), which is quickly dismutated into O_2_ and hydrogen peroxide (H_2_O_2_) by the iron superoxide dismutase (Fe-SOD). In the chloroplasts, the first ROS produced is singlet oxygen (^1^O_2_) due to light excitation of chlorophylls, which generates lasting chlorophyll triplet excited states that are quenched by carotenoids (Cejudo et al. 2021). Additionally, in an aqueous environment such as that within the cell, the presence of redox metals such as iron (Fe^2+^) and copper (Cu^2+^) allows for the occurrence of the Fenton reaction, which generates hydroxyl radicals (^·^OH) (Winterbourn 1995).

**Table 1:** Basic properties of reactive oxygen species (ROS) in a plant cell. Showing the foremost studied ROS in plant cell: triplet oxygen (^3^O_2_); superoxide (O_2_^·−^); hydroxyl radical (^·^OH); singlet oxygen (^1^O_2_) and hydrogen peroxide (H_2_O_2_).

	ROS	Structure	Life span (t_1/2_)	Migration distance	Main activities	References
bt	^**3**^ **O**_**2**_ Triplet oxigen		> 1 ms ground state	> 1 mm	Diradical, one-electron oxidant, respiration	(Pryor et al. 2006)
Free radicals	**O**_**2**_^·**−**^ Superoxide		1–4 ms	30 nm	Reacts with Fe-S proteins, dismutases to H_2_O_2_	(Fujii et al. 2022)
	^**·**^ **OH** Hydroxyl radical		1 ns	1 nm	Extremely reactive with DNA, RNA, lipids and proteins	(Dumanović et al. 2021)
Nonradicals	^**1**^ **O**_**2**_ Singlet oxygen		1–4 ms	30 nm	Oxidases lipids, proteins (attacking Trp, His, Tyr, Met and Cys residues) and G residues on DNA	(Fischer et al. 2013)
	**H**_**2**_ **O**_**2**_ Hydrogen peroxide		> 1 ms	> 1 mm	Reacts with proteins (attacking Cys and Met residues), heme proteins and DNA	(Hasanuzzaman et al. 2020)

Singlet oxygen (^1^O_2_) is a non-radical ROS that does not have an extra unpaired electron. Given that ^1^O_2_ has no spin constraints, it can interact almost indiscriminately with other biological molecules, including nucleic acids, proteins, and lipids (Dmitrieva et al. 2020; Yang et al. 2019). The first ^1^O_2_ targets are covalent double bonds, such as those in polyunsaturated fatty acids (e.g., oleic acid, linoleic acid) (Hajimohammadi et al. 2018) and some amino acids (e.g., histidine, cysteine, and methionine). Among the two types of lipid peroxidation (Girotti 1985), type I is triggered by free radicals with high redox potential, such as ^·^OH, whereas type II is mediated by ^1^O_2_ (nonradical) and is the main cause of photosynthetic tissue death due to photooxidative damage (Mor et al. 2014). Although ^1^O_2_ lifespan is very short (~ 40 ns), it is long enough to allow it to diffuse out of the chloroplast, interfere with signaling cascades in the cytosol and reach the cell wall, plasma membrane, and tonoplast (Fischer et al. 2013).

Superoxide (O_2_^·−^) and hydrogen peroxide (H_2_O_2_) are generated through common pathways, such as the electron transport chains of the mitochondrion and chloroplast, and the lipid β-oxidation pathway. Unlike singlet oxygen, superoxide is much less reactive and acts as both oxidant and reductant with great voracity for iron-sulphur clusters commonly present in enzymes (Fisher et al. 2016). It may also be the major prompter of tryptophan degradation pathway in peptides and proteins under stress (Carroll et al. 2018). Superoxide is, however, unlikely to act as a direct cellular signal because of its high instability and inability to diffuse through the membrane (Yang et al. 2019), even though there is convincing evidence that superoxide and other ROS could act as indirect signals by means of protein oxidative damage (Møller et al. 2011).

By contrast, hydrogen peroxide (H_2_O_2_) has particular properties for signaling, such as (i) a relatively long half-life (1 ms) (Mittler and Zilinskas 1991); (ii) the ability to diffuse through aquaporins embedded in membranes over great distances within the cell (Bienert et al. 2007); (iii) the ability of inhibiting important enzymes by oxidation of heme-thiol ligand to—SOH (Albertolle and Peter Guengerich 2018); (iv) mild and specific reactivity; and (v) the ability to be produced by several metabolic reactions via the reduction of other ROS, and to generate other ROS through the Fenton reaction (Sharma et al. 2012). H_2_O_2_ interacts with cysteine residues in proteins, which can lead to changes in the redox state of some polypeptides and trigger cell signaling cascades for cell proliferation, differentiation, and programmed cell death (Mittler 2017). The chloroplast is a major source of H_2_O_2_ in the cell and, therefore, H_2_O_2_ is an important signal during photosynthesis (Cejudo et al. 2021; Pilon et al. 2011).

### Interactions between anthocyanins and ROS

Numerous abiotic stresses and even regular metabolism can promote an increase in ROS production (Hasanuzzaman et al. 2020; Huang et al. 2019). The balance between ROS production and elimination must be strictly maintained in the cell at any moment to avoid lethal damage. One way to achieve this homeostasis is through the counterbalance with antioxidants, such as flavonoids, of which anthocyanins may constitute a significant portion. A key issue is the potential for physical interaction between anthocyanins and ROS, which has hitherto not been addressed in-depth (except for flavonoids in general) (Agati et al. 2020, 2021; Ferreyra et al. 2021). Indeed, there is evidence showing in vitro antioxidant activity as previously discussed, and usually the lack of flavonols biosynthesis is associated with ROS homeostasis imbalance (Chapman and Muday 2021; Muhlemann et al. 2018; Silva-Navas et al. 2016). Nevertheless, the mechanism by which anthocyanins, and not flavonols, interacts with ROS to promote their scavenging is poorly understood.

Anthocyanins are synthesized in the cytosol and transported for storage in the vacuole via three different pathways: glutathione S-transferases (GSTs) (Sun et al. 2012), multidrug and toxic compound extrusion (MATE) (Gomez et al. 2009) and ATP-binding cassette (ABC) transporters (Behrens et al. 2019). The following scenario is proposed. The cytosol flavonol pool may be regulated and replenished by anthocyanins stored in the vacuole, and when ROS levels increase, the flavonol pool is depleted. Vacuolar glucosidase then breaks down the anthocyanins (Wang et al. 2011) and aglycones are transferred from the vacuole to help regulate ROS homeostasis in the cytosol, either by immediate interaction as a H-donor and single electron transfer or by the activity of enzymes such as peroxidases (Liu et al. 2018; Oren-Shamir 2009; Tena et al., 2020). However, this is quite unclear, even though the association between flavonoids (flavonols and anthocyanins) content and ROS level is known to be antagonistic.

As discussed previously, flavonoid biosynthesis is energetically costly and finely controlled as a direct or indirect response to changes in ROS content. Therefore, the biotechnological manipulation of anthocyanin content in crops (e.g. Zhang et al. 2014) must be carefully balanced with the normal function of ROS signaling. To explore this notion, we briefly consider key points of intersection between anthocyanins and ROS in plant development, namely in the processes of seed germination, root development, pollen tube growth and senescence.

### Seed germination

Germination starts with tissue hydration and an increase in respiration that leads to a rise in ROS production by respiration burst oxidative homologue (RBOH) proteins (Moles et al. 2019; Muller et al. 2009). The process elevates the content of several ROS, including H_2_O_2_, O_2_^·**−**^ and ^·^OH·H_2_O_2_ disturbs seed dormancy in multiple ways, especially by enhancing ABA catabolism and promoting biosynthesis of gibberellins (GAs) (Liu et al. 2010; Wang et al. 2019). Interestingly, anthocyanins affect germination by counteracting ROS. Decreased anthocyanin synthesis led to a decrease in seed dormancy (McCarty et al. 1989; Zhao et al. 2019). On the other hand, stress that intensifies ROS production above a certain threshold that underpins breakage of seed dormancy, can impair germination and seedling establishment rather than favouring it (Jeevan Kumar et al. 2015). Differential dormancy exists in seeds with different colors in the same species (Zhao et al. 2019). The *Leymus chinensis* transcription factor *LcbHLH92* is negatively correlated with anthocyanin/proanthocyanidin-specific genes, and its overexpression in Arabidopsis significantly inhibited the transcript levels of *DFR* and *ANS* genes in leaves and seeds, resulting in yellow seeds with higher germination rates compared to the wild-type brown seeds (Zhao et al. 2019).

### Root development

Besides their obvious functions, roots are also a frontline stress-sensing organ that relays information to the shoot (Burko et al. 2020; Glanz-Idan et al. 2020). For instance, accumulation of heavy metals promotes radical superoxide (O_2_^·−^), hydroxyl radicals (^·^OH) and hydrogen peroxide (H_2_O_2_) production, leading to toxicity symptoms (Asati et al. 2016). In chickpea (*Cicer arietinum*) roots under hypoxia, anthocyanin biosynthesis was associated with an increase in ROS (Nazari et al. 2019). Nonetheless, ROS are also important for root development. The tomato (*Solanum lycopersicum*) mutant *anthocyanin reduced* (*are*) is a knockout for the *FLAVONOID 3-HYDROXYLASE* (*F3H*) gene. It accumulates low anthocyanin levels and concomitantly shows higher ROS content in roots and increased root hair formation compared to the wild type (Maloney et al. 2014). The influence of flavonols and anthocyanins on lateral root development may be facilitated by their inhibition of auxin transported since decreased flavonols content in the *are* mutant resulted in greater auxin flux away from the maturation zone (Maloney et al. 2014).

Another possibility is that plants may use flavonoids as carbon skeleton reserves, suggesting that phenolics in general, including anthocyanins, may be a carbon sink for excess photosynthetic carbon (Lo Piccolo et al. 2020). This is directly related with anthocyanin accumulation in leaves, but the role of anthocyanin accumulation in roots for energy storage is yet to be elucidated. In Arabidopsis, the anthocyanin precursor kaempferol acts by reducing superoxide and ROS concentration, i.e., as a negative regulator of ROS-stimulated lateral root emergence (Chapman and Muday 2021). Furthermore, flavonoids are related to root light avoidance in plants, that upon perceiving light, flavonols are directed to the lighted region in the elongation zone, which changes the internal gradient of superoxide and auxin in the copula, promoting root bending to avoid light (Silva-Navas et al. 2016).

### Pollen tube growth

Sexual reproduction of plants requires long-distance growth of the pollen tube for fertilization of the female gametophyte. Recent reports suggest that this process is regulated by antagonic interactions between flavonols and ROS (O_2_^·−^ and ^·^OH) and hydrogen peroxide (H_2_O_2_), whereby flavonols play an important role in keeping ROS homeostasis, preventing it from reaching the inhibitory level during heat stress (Chen et al. 2021; Muhlemann et al. 2018). Additionally, the anthocyanin reduced (*are*) tomato mutant has reduced flavonol accumulation in pollen grains and tubes, associated with impaired pollen viability, germination, tube growth, and tube integrity, resulting in reduced seed set (Muhlemann et al. 2018). These effects were reverted by antioxidant treatment and revealed that flavonol metabolites regulate plant sexual reproduction at both normal and elevated temperatures by maintaining ROS homeostasis.

### Senescence

Senescence is a highly regulated ontogenetic process that leads to ROS accumulation and ultimately cell death (Thomas et al. 2003). In leaves, it is accompanied by degradation of chlorophylls and anthocyanin synthesis (Merzlyak et al. 2008; Vangelisti et al. 2020). During senescence, carbon export from source to sink is reduced, leading to hexose accumulation (Chen et al. 2022). This increase in sugar content induces the biosynthesis of phenylpropanoids, including anthocyanins (Nardozza et al. 2020). A proposed mechanism states anthocyanin biosynthesis represents an alternative carbon sink that limits sugar hyperaccumulation to avoid feedback inhibition of the photosynthetic machinery, including a reduction of ribulose-1,5-biphosphate carboxylase–oxygenase (Rubisco) expression (Lastdrager et al. 2014). The induction of anthocyanin accumulation by ROS signaling during senescence should also account for the negative effect of flavonoids on auxin transport. A number of synthetic compounds have been shown to block the process of auxin transport by inhibition of the auxin efflux carrier complex. These synthetic auxin transport inhibitors may act by mimicking endogenous molecules. Flavonoids have been suggested to be auxin transport inhibitors based on their in vitro activity (Brown et al., 2001). The hypothesis that flavonoids regulate auxin transport in vivo was tested in Arabidopsis by comparing wild-type (WT) and *transparent testa 4* (*tt4*) plants with a mutation in the gene encoding the first enzyme in flavonoid biosynthesis, chalcone synthase. In a comparison between *tt4* and WT plants, phenotypic differences were observed, including three times as many secondary inflorescence stems, reduced plant height, decreased stem diameter, and increased secondary root development. Growth of WT Arabidopsis plants on naringenin, a biosynthetic precursor to those flavonoids with auxin transport inhibitor activity in vitro, leads to a reduction in root growth and gravitropism, similar to the effects of synthetic auxin transport inhibitors. Analyses of auxin transport in the inflorescence and hypocotyl of independent *tt4* alleles indicate that auxin transport is elevated in plants with a *tt4* mutation. In hypocotyls of *tt4*, this elevated transport is reversed when flavonoids are synthesized by growth of plants on the flavonoid precursor, naringenin. These results are consistent with a role for flavonoids as endogenous regulators of auxin transport (Brown et al. 2001), since this hormone delays senescence by acting antagonistically to ethylene (Guo et al. 2021).

### Concluding remarks

Anthocyanins play an important role in the induction of morpho-physiological modifications to facilitate plant adaptation to environmental stresses. High anthocyanin content provides protection against excessive irradiance by both attenuation of light absorption by the leaf and ROS-scavenging activity. However, the relative importance of anthocyanins in these responses remains controversial, mainly because of their vacuolar localization, which is spatially removed from the primary sites of ROS production in other organelles. It is also clear that stress conditions, either biotic or abiotic, lead to high ROS accumulation. ROS can react with and damage cellular components, but they also participate in cell signaling cascades. Many general stress response genes are regulated by signaling pathways that uses ROS as indirect messengers and different ROS formed in the same cell compartment can result in different intermediate signaling molecules that regulate specific gene clusters, leading to a direct response related to the stress condition at play.

It is essential, therefore, to assess the impact of anthocyanins (instead of flavonols in general) accumulation on abiotic stress resistances. In many instances, anthocyanin biosynthesis is induced as a response to attacks of pathogens (e.g., (Kangatharalingam et al. 2002) and insects (Li et al. 2019a, b; Schaefer and Rolshausen 2006). Even though a relationship between anthocyanin accumulation and abiotic stress appears to exist, the underlying mechanisms of this response and the impact on the redox status of the plant still need to be systematically explored.

The impact of anthocyanin overaccumulation on crop yield has not been fully established. Some attention and systematic analyses assessing how anthocyanins and other antioxidants may affect cell signaling and plant phenotypes will be helpful to enable breeding pipelines to create anthocyanin-enhanced crop varieties without adverse effects (e.g. low germination rates, undesirable developmental phenotypes, lower photosynthesis rates) (Sestari et al. 2014). In tomato, a promoter replacement line overexpressing *ANTHOCYANIN1* (*ANT1*) showed reduced leaf thickness and impaired side branching, with a concomitant penalty in fruit yield (Cerqueira et al. 2022). A more in-depth assessment of the impact of anthocyanin accumulation on yield comparing cyanic and isogenic non-cyanic genotypes within a redox physiology perspective could be revealing both at the fundamental and applied levels and may lead to greater understanding as to the subtle interplay of this team of rivals.

## Data Availability

All data supporting the findings of the present study are available within the paper.
